# Cochrane Eyes and Vision: systematic reviews on myopia

**Published:** 2019-05-13

**Authors:** Jennifer Evans

**Affiliations:** 1 Assistant Professor (Epidemology): London School of Hygiene & Tropical Medicine, London, UK.

**Systematic reviews offer high quality, evidence-based guidance to health professionals. These reviews address myopia and its complications.**

**Figure:**
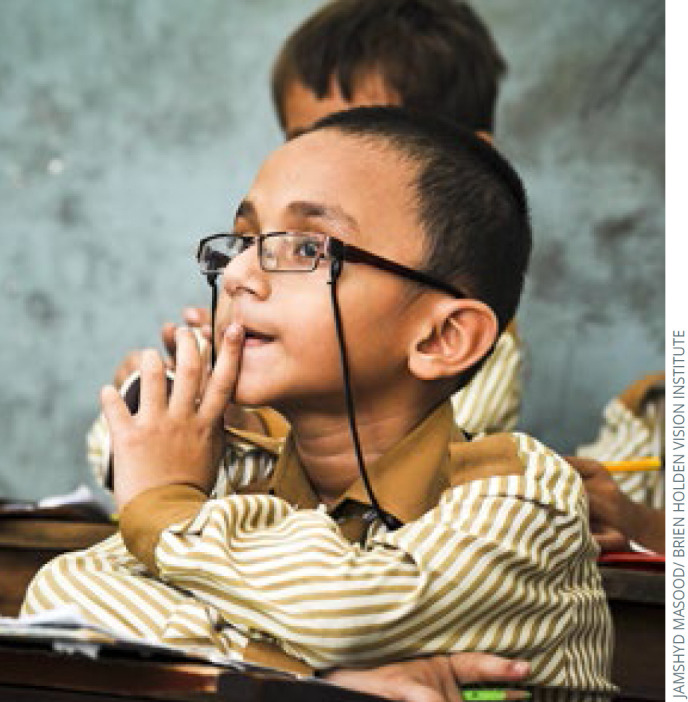
Providing free spectacles improves the number of children who have and wear their spectacles. PAKISTAN

Cochrane Eyes and Vision (CEV) is an international network of individuals working to prepare, maintain and promote access to systematic reviews of interventions to treat or prevent eye diseases or visual impairment, and reviews of the accuracy of diagnostic tests. Systematic reviews are summaries of the best available evidence and are designed to answer a specific research question. The reviews featured here are published in the Cochrane Library, which is available free of charge in low- and middle-income countries via the Hinari Programme. **www.who.int/hinari**

## 1 Interventions to slow progression of myopia in children

**www.cochranelibrary.com/cdsr/doi/10.1002/14651858.CD004916.pub3**

**Date:** December 2011. Update due in 2019.

**Key findings:** Anti-muscarinic topical medication slows the progression of myopia in children. Adverse effects include light sensitivity and near blur.

## 2 Vision screening for correctible visual acuity deficits in school-age children and adolescents

**www.cochranelibrary.com/cdsr/doi/10.1002/14651858.CD005023.pub3**

**Date:** February 2018

**Key findings:** Vision screening plus provision of free spectacles improves the number of children who have and wear the spectacles they need compared with providing a prescription only.

## 3 Laser photocoagulation for choroidal neovascularisation in pathologic myopia

**www.cochranelibrary.com/cdsr/doi/10.1002/14651858.CD004765.pub2/**

**Date:** March 2007

**Key findings:** The effect of laser photocoagulation to treat choroidal neovascularisation due to myopia is uncertain. Adverse effects include enlargement of the atrophic laser scar which is potentially vision threatening.

## 4 Anti-vascular endothelial growth factor for choroidal neovascularisation in people with pathological myopia

**www.cochranelibrary.com/cdsr/doi/10.1002/14651858.CD011160.pub2**

**Date:** December 2016

**Key findings:** Low and moderate-certainty evidence that people receiving anti-vascular endothelial growth factor have a better outcome in terms of visual acuity compared with no treatment, photodynamic therapy or laser. Adverse effects occurred rarely.

